# Alkanols inhibit voltage-gated K^+^ channels via a distinct gating modifying mechanism that prevents gate opening

**DOI:** 10.1038/srep17402

**Published:** 2015-11-30

**Authors:** Evelyn Martínez-Morales, Ivan Kopljar, Dirk J. Snyders, Alain J. Labro

**Affiliations:** 1 Laboratory for Molecular Biophysics, Physiology and Pharmacology, Department of Biomedical Sciences, University of Antwerp, Antwerp, 2610, Belgium

## Abstract

Alkanols are small aliphatic compounds that inhibit voltage-gated K^+^ (K_v_) channels through a yet unresolved gating mechanism. K_v_ channels detect changes in the membrane potential with their voltage-sensing domains (VSDs) that reorient and generate a transient gating current. Both 1-Butanol (1-BuOH) and 1-Hexanol (1-HeOH) inhibited the ionic currents of the *Shaker* K_v_ channel in a concentration dependent manner with an IC_50_ value of approximately 50 mM and 3 mM, respectively. Using the non-conducting *Shaker*-W434F mutant, we found that both alkanols immobilized approximately 10% of the gating charge and accelerated the deactivating gating currents simultaneously with ionic current inhibition. Thus, alkanols prevent the final VSD movement(s) that is associated with channel gate opening. Applying 1-BuOH and 1-HeOH to the *Shaker*-P475A mutant, in which the final gating transition is isolated from earlier VSD movements, strengthened that neither alkanol affected the early VSD movements. Drug competition experiments showed that alkanols do not share the binding site of 4-aminopyridine, a drug that exerts a similar effect at the gating current level. Thus, alkanols inhibit *Shaker*-type K_v_ channels via a unique gating modifying mechanism that stabilizes the channel in its non-conducting activated state.

Alkanols (or 1-alcohols) are small volatile aliphatic compounds that partition rapidly across the plasma membrane and have the potential to induce anesthesia at high doses^1^. Alkanols have been shown to target both cytoplasmic and plasma membrane proteins^2^,^3^, including voltage-gated K^+^ (K_v_) channels^4^. K_v_ channels play an important role in cellular excitability as they constitute the cell’s repolarizing power; they shape the action potential duration and help setting the threshold for initiating one.

K_v_ channels are assembled from four α-subunits, each containing six transmembrane segments (S1–S6) whereby the S5–S6 segments create the K^+^ pore^5^. K^+^ flow through this pore is controlled by a channel gate that is located in the lower carboxyl-terminal part of S6 (S6_c_)^6^. Via an electromechanical coupling, composed of the S4–S5 linker and S6_c_, opening and closure of the channel gate is controlled by the four voltage-sensing domains (VSD) that consist of the S1–S4 segments. Upon changes in the membrane potential, the positively charged residues on the S4 segment (gating charges) move across the plasma membrane generating a transient ‘gating current’ (I_G_)^7^,^8^. In the generally accepted gating scheme for *Shaker*-type K_v_ channels, the four VSDs move in a largely independent way from their inward facing rested state to their outward facing activated configuration^9^,^10^,^11^. This transition(s) carries approximately 90% of the total gating charge but does not open the channel gate. Once all four VSDs have reached their activated state, channel gate opening proceeds in a subunit-concerted manner which is accompanied by moving the last 10% of the gating charge^12^,^13^,^14^. Furthermore, channel gate opening stabilizes the VSD in its outward facing activated state and is manifested in slower VSD deactivation kinetics^10^,^15^.

Analyzing the sensitivity of different K_v_ channels to alkanols revealed an inter-species difference wherein the K_v_ channels from the fruit fly *drosophila melanogaster* displayed a higher affinity than their mammalian orthologs^16^,^17^. The higher alkanol sensitivity of the *drosophila Shaw*2 channel could be transplanted onto its mammalian K_v_3.4 counterpart by exchanging the S4–S5 linker^18^. Site-specific residue substitution studies further supported that the S4–S5 linker forms with possible contribution of S6_c_ a key determinant in channel inhibition by alkanols^4^,^19^,^20^,^21^,^22^. To elucidate alkanols’ mechanism of action, we performed detailed gating current analysis of the *drosophila Shaker* K_v_ channel and show that 1-Butanol (1-BuOH) and 1-Hexanol (1-HeOH) stabilize the channel in the non-conducting activated state, which results in a 10% reduction in gating charge movement and an accelerated VSD deactivation. Although this behavior was reminiscent to the effect of 4-aminopyridine (4-AP)^14^,^23^, alkanols act via a distinct binding site for preventing the *Shaker* K_v_ channel of passing the final subunit-concerted transition leading to channel opening.

## Results

### Concentration-dependent inhibition of Shaker K_v_ channel by 1-BuOH and 1-HeOH

The *drosophila Shaw2 *K_v_ channel was identified to display the highest sensitivity for alkanols and has therefore been the subject to study the mechanism of their action^4^,^16^,^19^,^21^. The *drosophila Shaker* K_v_ channel was also inhibited by alkanols but compared to *Shaw2* it displayed a lower sensitivity^17^. Since its cloning, the *Shaker* K_v_ channel became rapidly the prototypical K_v_ channel for structure-function studies and most of the current knowledge on the operation of the VSDs, the electromechanical coupling and the channel gate is based on studies in this channel. Thus, despite its lower sensitivity, the available knowledge on the gating mechanism is an advantage of the *Shaker* K_v_ channel for determining the mechanism of channel inhibition by alkanols.

**Figure 1:**
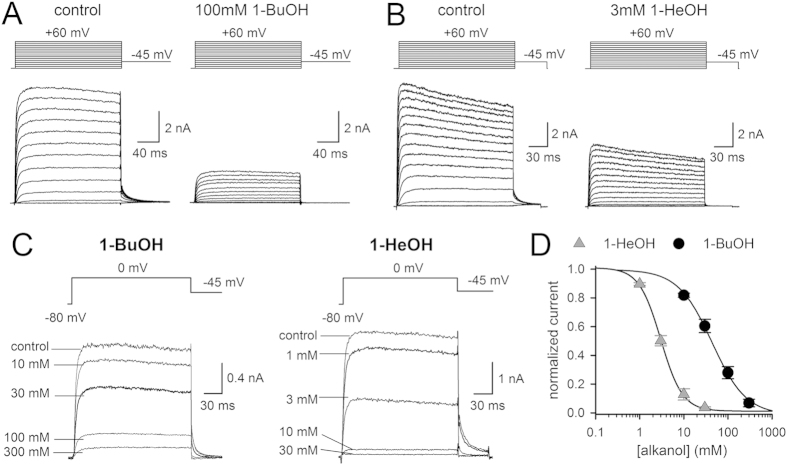
Inhibition of *Shaker*-IR by 1-BuOH and 1-HeOH. (**A**) Representative I_K_ recordings of *Shaker*-IR in control condition (left) and in presence of 100 mM 1-BuOH (right) elicited by applying depolarization steps from a -80* *mV holding potential (pulse protocols are shown on top). (**B**) I_K_ recordings of *Shaker*-IR obtained in control conditions (left) and in presence of 3* *mM 1-HeOH (right). (**C**) Steady-state I_K_ recordings (elicited with a voltage step from -80* *mV to 0* *mV) upon wash-in of different concentrations of 1-BuOH (left) and 1-HeOH (right). Establishment of channel inhibition was monitored by repetitive pulsing to 0* *mV. (**D**) Concentration-response curves obtained by plotting the normalized steady-state I_K_ amplitude at 0* *mV, determined from I_K_ recordings as shown in panel C, as a function of 1-BuOH (circles, *n* = 5) or 1-HeOH (triangles, *n* = 7) concentration. Solid lines represent the average fit with a Hill function.

**Figure 2:**
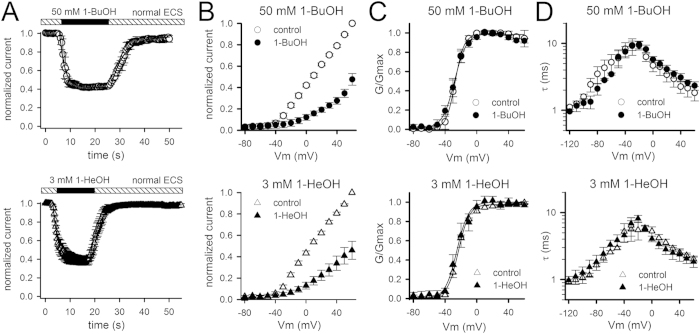
Alkanols inhibited I_K_ without affecting the kinetics. (**A**) Monitoring the inhibition in I_K_ during application of 50* *mM 1-BuOH (top panel) or 3* *mM 1-HeOH (bottom panel) indicated that the I_K_ inhibition developed rapidly with a time constant of 5.2 ± 1.2 s (*n* = 8) and 3.7 ± 0.7 s (*n* = 9) for 1-BuOH and 1-HeOH respectively. The I_K_ inhibition was fully reversible upon wash-out of both alkanols and the current recovery was relatively fast yielding time constants of 7.6 ± 2.1 s (*n* = 8) and 4.8 ± 1.7 s (*n* = 9) for 1-BuOH and 1-HeOH respectively. (**B**) Normalized peak current versus voltage relationships, obtained from pulse protocols shown in Fig. 1A, in control conditions (open symbols) and presence of 50* *mM 1-BuOH (top panel, *n* = 5) or 3* *mM 1-HeOH (bottom panel, *n* = 6). (**C**) Normalized conduction versus voltage GV curves in control conditions (open symbols) and presence of 50* *mM 1-BuOH (top panel, *n* = 5) or 3* *mM 1-HeOH (bottom panel, *n* = 6). Solid lines represent the average fit with a Boltzmann equation (V_1/2_ and slope factor values are provided in Table 1). (**D**) Time constants of I_K_ activation (τI_Kac_) and deactivation (τI_Kdeac_) in control conditions (open symbols) and in presence of 50* *mM 1-BuOH (top panel, *n* = 7) or 3* *mM 1-HeOH (bottom panel, *n* = 8).

Alkanols are classified in short chain (up to 5 carbonyls, C1 to C5) or long chain (C6 – C22) 1-alcohols^24^. In this study, 1-BuOH and 1-HeOH were chosen as representative compounds of a short and long chain alkanol. Their effect was tested on both the ionic (I_K_) and gating (I_G_) currents of the fast (N-type) inactivation removed *Shaker*-IR channel. At the I_K_ level, *Shaker-*IR was inhibited by both 1-BuOH and 1-HeOH in a concentration-dependent manner (Fig. 1A–C). For 1-BuOH a concentration-response curve was obtained with an IC_50_ value of 51.8 ± 5.9 mM (*n* = 5) and a Hill coefficient of 0.92 ± 0.04 (Fig. 1D). 1-HeOH had a slightly higher affinity and yielded a concentration-response curve with an IC_50_ value of 2.7 ± 0.2 mM (*n* = 7) and a Hill coefficient of 1.13 ± 0.22 (Fig. 1D). Monitoring the development of I_K_ inhibition and analyzing the remaining steady-state I_K_ amplitude upon application of 50 mM 1-BuOH or 3 mM 1-HeOH (IC_50_ concentrations) indicated that: (1) the I_K_ inhibition developed rapidly and was fully reversible upon wash-out of both alkanols, and (2) both alkanols did not induce major alterations in the voltage dependence of channel opening nor the time constants of channel activation (τI_Kac_) and deactivation (τI_Kdeac_) (Fig. 2 and Table 1). An apparent channel inactivation behavior or rising phase in the deactivating (I_Kdeac_) tail current (i.e. a hooked tail), which are typical hallmarks for an open channel blocker, were not observed (Fig. 1A–C). Thus, 1-BuOH and 1-HeOH inhibited the I_K_ amplitude without affecting the kinetics, and both compounds achieved this through a mechanism most likely different from open channel block, as proposed previously^4^.

### 1-BuOH and 1-HeOH accelerate VSD deactivation and immobilize approximately 10% of the gating charge

**Figure 3:**
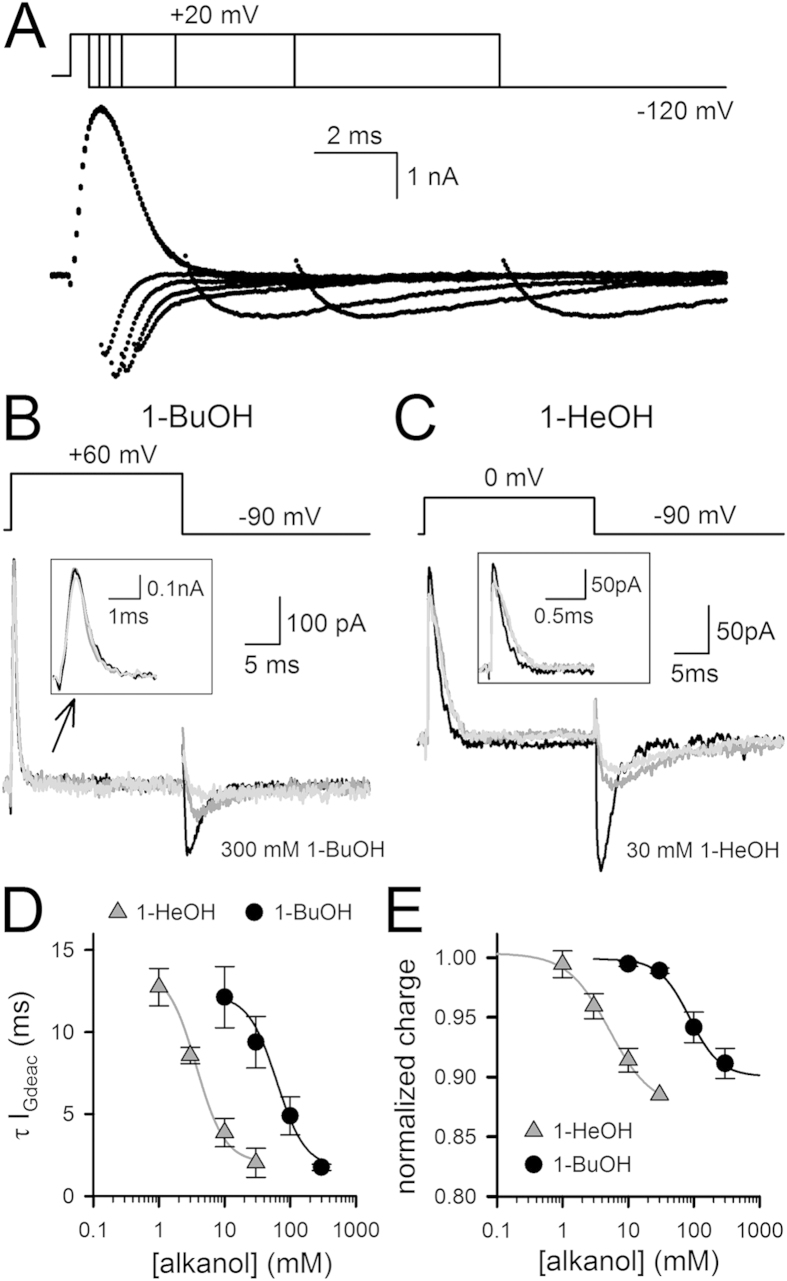
Impact of alkanols on I_G_ recordings of *Shaker*-IR-W434F. (**A**) Representative I_G_ recordings of *Shaker*-IR-W434F recorded in control conditions with the pulse protocol shown on top. Note that prolonging the depolarization at +20* *mV gradually slowed down I_Gdeac_ upon repolarization to −120* *mV. (**B**) Superposition of *Shaker*-IR-W434F steady-state I_G_ recordings in control condition (gray) and in presence of 100 mM (dark gray) and 300* *mM (black) 1-BuOH. Inset shows a scale up view of I_Gac_. Note the gradual acceleration in I_Gdeac_ upon application of higher concentrations of 1-BuOH. (**C**) Superposition of steady-state I_G_ recordings in control condition (gray) and in presence of 3* *mM (dark gray) and 30* *mM (black) 1-HeOH. (**D**) Concentration-response curves obtained by plotting the weighted τI_Gdeac_ at −90* *mV (obtained from I_Gdeac_ recordings shown in panel A and B) as a function of 1-BuOH (circles, *n* = 10) or 1-HeOH (triangles, *n* = 6) concentration. (**E**) Concentration-response curves obtained by plotting the normalized charge movement, which was determined by integrating the steady-state I_Gac_ recordings and normalizing the calculated charge to the total charge moved in control condition, as a function of alkanol concentration.

The I_K_ measurements only report on the final opening of the channel gate, which is an end state in the activation pathway from closed to open. From I_G_ analysis it has been reported that the VSD traverses at least one non-conducting activated state before the channel gate opens. Channel gate opening subsequently slows down VSD deactivation^10^,^15^, which can be visualized by gradually prolonging the duration of the depolarizing pre-pulse (Fig. 3A). Thus, to assess whether 1-BuOH and 1-HeOH affect transitions early in the activation pathway, i.e. before the channel gate opened, we tested the effect of both compounds on the I_G_ recordings of the non-conducting *Shaker*-IR pore mutant W434F^25^. During wash-in of both 1-BuOH and 1-HeOH we noted a concentration-dependent acceleration of the deactivating (I_Gdeac_) gating currents (Fig. 3B,C). Plotting the time constant of VSD deactivation (τI_Gdeac_, obtained by fitting the decaying phase of I_Gdeac_) as a function of 1-BuOH or 1-HeOH concentration yielded concentration-response curves with IC_50_ values of 67 ± 1 mM (*n* = 10) and 3.0 ± 0.4 mM (*n* = 6), and Hill coefficients of 1.3 ± 0.4 and 1.6 ± 0.3, respectively (Fig. 3D).

To examine whether this acceleration in τI_Gdeac_ was associated with a reduction in gating charge movement, we integrated the activating (I_Gac_) gating currents (elicited during the depolarizing test pulse) after reaching steady-state modification of the τI_Gdeac_ kinetics. This analysis indicated that there was an alkanol-dependent reduction in gating charge movement concomitantly with the acceleration in τI_Gdeac_. The reduction in total gating charge as a function of alkanol concentration yielded for 1-BuOH and 1-HeOH concentration-response curves with IC_50_ values of 88 ± 2 mM (*n* = 10) and 13.8 ± 1.6 mM (*n* = 6), and Hill coefficients of 1.5 ± 0.2 and 1.5 ± 0.2, respectively (Fig. 3E). Based on these concentration-response curves, the maximal reduction in charge movement amounted to approximately 10% and 12% upon application of 300 mM 1-BuOH and 30 mM 1-HeOH, respectively.

**Figure 4:**
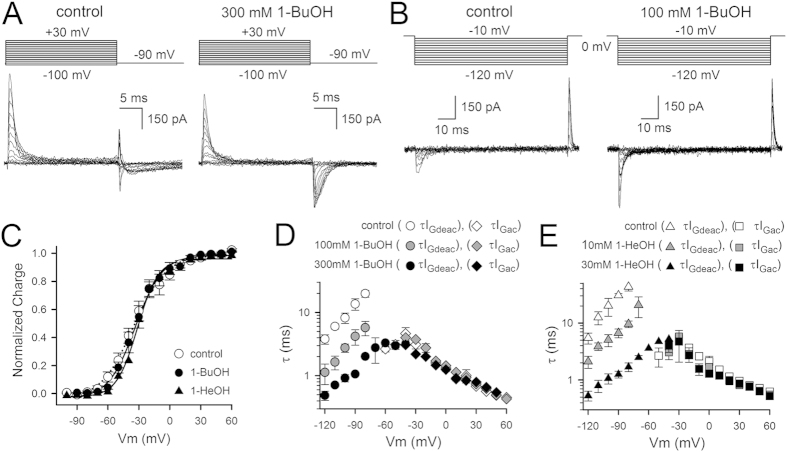
Biophysical properties of *Shaker*-IR-W434F upon alkanol application. (**A**) Representative I_Gac_ recordings of *Shaker*-IR-W434F in control condition (left) and in presence of 300* *mM 1-BuOH (right) elicited using the pulse protocols shown on top. (**B**) Representative I_Gdeac_ recordings elicited with the deactivation pulse protocols shown on top; in control conditions (left) and in presence of 100 mM 1-BuOH (right). Inter-sweep holding potential was −90* *mV and the depolarizing pre- and post-pulse to 0* *mV were 15* *ms in duration. (**C**) Charge vs. voltage QV curves in control condition (white circles, *n* = 10) and in presence of 300* *mM 1-BuOH (black circles, *n* = 5) or 30* *mM 1-HeOH (black triangles, *n* = 4) were created by plotting the normalized charge (obtained from integrating I_Gac_ recordings from pulse protocols shown in panel A) as a function of voltage. Curves shown are the average fit to a Boltzmann equation. (**D**) Time constants of VSD activation (τI_Gac_) in control condition (white diamonds, *n* = 8) and in presence of 100* *mM (gray diamonds, *n* = 3) or 300* *mM (black diamonds, *n* = 5) 1-BuOH. For VSD deactivation the weighted τI_Gdeac_ kinetics are shown. Note the gradual acceleration in τI_Gdeac_ between control (white circles), 100* *mM 1-BuOH (gray circles) and 300* *mM 1-BuOH (black circles). (**E**) Panel shows the voltage-dependent τI_Gac_ kinetics in control condition (white squares, *n* = 7) and in presence of 10* *mM (gray squares, *n* = 3) or 30* *mM (black squares, *n* = 4) 1-HeOH. Similar to 1-BuOH the τI_Gdeac_ kinetics accelerated in presence of 10* *mM (gray triangles, *n* = 3) and 30* *mM 1-HeOH (black triangles, *n* = 4), control conditions (white triangles).

To determine the kinetics and voltage dependence of VSD activation, we applied incremental depolarizing voltage steps starting from a constant hyperpolarized initial voltage (activation protocol, Fig. 4A). To characterize VSD deactivation adequately, a deactivation pulse protocol was used (Fig. 4B). Integrating the I_Gac_ recordings, obtained in control conditions and after steady-state 1-BuOH and 1-HeOH modification, yielded charge vs. voltage QV curves (Fig. 4C). Interestingly, the QV curves determined in presence of 1-BuOH or 1-HeOH displayed V_1/2_ and slope factor values similar as in control condition (Table 1). This indicated that neither alkanol affected the voltage dependence of the remaining gating charge movement. As noted during the wash-in protocol (Fig. 3B,C), both alkanols accelerated τI_Gdeac_ without markedly altering the I_Gac_ kinetics (τI_Gac_, Fig. 4D,E). Thus, both alkanols accelerated τI_Gdeac_ and immobilized approximately 10% of the gating charge movement but did not affect the voltage dependence of the early VSD movements. These observations indicated that in presence of 1-BuOH or 1-HeOH the *Shaker* channel is able to reach the non-conducting activated state but it cannot pass the subunit-cooperative step leading to channel gate opening. Accordingly, the τI_Gdeac_ values in presence of saturating alkanol concentrations should corresponded to τI_Gdeac_ in control conditions when the activating pre-pulse is very short and channels only reach the non-conducting activated state. In control conditions τI_Gdeac_ amounted at −120 mV to 0.32 ± 0.03 ms (*n* = 6) upon a brief 0.5 ms depolarization, determined from pulse protocols shown in Fig. 3A. In presence of 300 mM 1-BuOH or 30 mM 1-HeOH τI_Gdeac_ at −120 mV were 0.48 ± 0.08 ms (*n* = 4) and 0.53 ± 0.10 ms (*n* = 4) respectively (Fig. 4D,E), which are indeed similar to the value in control conditions.

### 1-alkanols and 4-AP have different binding sites but immobilize the same gating charge component

**Figure 5:**
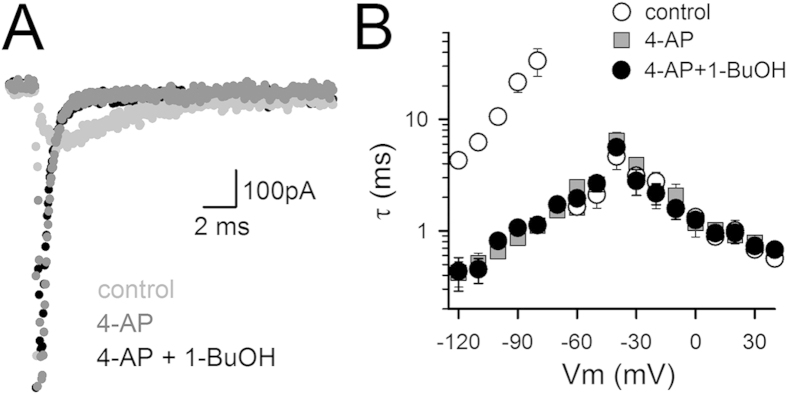
1-BuOH and 4-AP immobilize the same gating charge component. (**A**) Superposition of steady-state I_Gdeac_ recordings of *Shaker*-IR-W434F, elicited during a repolarizing step to -120* *mV upon a 50* *ms depolarization at 0* *mV, in control condition (light gray), in presence of 1* *mM 4-AP (dark gray), and in presence of 1* *mM 4-AP plus 300* *mM 1-BuOH (black). Note that the mixture of 4-AP plus 1-BuOH did not result in an extra acceleration of I_Gdeac_ decay or an extra reduction in gating charge movement. (**B**) Panel shows τI_Gac_ and τI_Gdeac_ in control condition (white circles, *n* = 6), in presence of 4-AP (dark gray squares, *n* = 5), and 4-AP plus 1-BuOH mixture (black circles, *n* = 6). Both drug conditions resulted in a similar acceleration of τI_Gdeac_ without affecting τI_Gac_ markedly.

The impact of 1-BuOH and 1-HeOH on the I_G_ recordings of the *Shaker*-IR-W434F channel was reminiscent of the effect of 4-AP that prevents the channels from passing the late subunit-cooperative step of channel gate opening, resulting in a similar 10% reduction in gating charge movement^14^. To assess if 4-AP and 1-BuOH immobilized the same gating charge component, we determined the reduction in gating charge movement using a mixture of 1 mM 4-AP and 300 mM 1-BuOH, which for both compounds are saturating concentrations. First, we applied 1 mM 4-AP that resulted in an approximately 10% loss of gating charge movement and an acceleration of τI_Gdeac_, as has been described before^14^,^23^. After establishing a steady-state 4-AP effect, we applied 300 mM 1-BuOH in the continued presence of 1 mM 4-AP. The addition of 1-BuOH did not result in an extra reduction of gating charge movement or further acceleration of the I_Gdeac_ kinetics (Fig. 5). This indicated that both compounds affected the same gating charge component and further supported that alkanols stabilize the channel in the non-conducting activated state similar to 4-AP.

**Figure 6:**
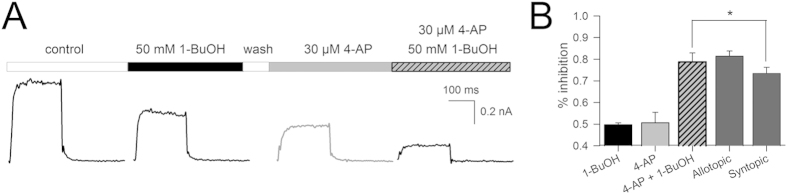
1-BuOH and 4-AP do not compete for inhibiting *Shaker*-IR. (**A**) Sequentially recorded I_K_ of *Shaker*-IR in control condition and after steady-state inhibition by 50* *mM 1-BuOH and 30 μM 4-AP. Finally, instead of washing the 30 μM 4-AP out, a mixture of 30 μM 4-AP plus 50* *mM 1-BuOH was added and the amount of I_K_ inhibition was determined. (**B**) Bar chart shows the average reduction in I_K_ ± S.E.M. (*n* = 7) after applying 50* *mM 1-BuOH, 30 μM 4-AP and the mixture of both compounds (30 μM 4-AP plus 50* *mM 1-BuOH). The percentage of I_K_ inhibition was calculated by normalizing the steady-state I_K_ in presence of drug to the I_K_ amplitude in control conditions. The expected reduction in I_K_ for an allotopic and syntopic model was calculated as described in Material and Methods. Note, the experimentally obtained value differed only statistically from the predicted value of a syntopic model (*p < 0.05).

Although alkanols and 4-AP exert a similar effect at the gating current level, they may act through different binding sites. Whereas the binding site of 4-AP partially overlaps with that of internal pore blockers^26^,^27^, alkanols have been proposed to target the electromechanical coupling that is located outside the K^+^ pore. To test whether 4-AP and 1-BuOH have structurally different binding sites, we performed drug competition experiments using IC_50_ concentrations of 4-AP (30 μM) and 1-BuOH (50 mM). After establishing approximately 50% steady-state I_K_ inhibition with 4-AP, we applied a mixture of 30 μM 4-AP and 50 mM 1-BuOH. This mixture resulted in 78.7 ± 4.1% (*n* = 7) inhibition of I_K_ (Fig. 6), thus yielding an additional inhibition of 29% in I_K_ amplitude compared to each compound separately.

To evaluate whether 4-AP and 1-BuOH competed, the expected inhibition of the mixture was calculated using a syntopic (both compounds compete) or an allotopic (no competition) model^28^. Using an allotopic model and the experimentally determined inhibition of each compound separately, the predicted inhibition of the mixture was 81.4 ± 2.4% (*n* = 7). With a syntopic model the predicted inhibition was 73.4 ± 2.8% (*n* = 7). Because the experimentally determined inhibition (78.7%) differed only statistically (p < 0.05) from the predicted value of the syntopic model (Fig. 6), our data matched best an allotopic model indicating that there was no competition between both compounds.

### 1-BuOH and 1-HeOH activate the Shaker-IR-P475A mutant by accelerating channel opening

A previous study reported that substituting a highly conserved proline residue in the S6_c_ of the *Shaw2* channel (the second proline of a highly conserved PXP motif within the S6_c_ of K_v_ channels) by a neutral amino acid such as alanine inverted the effect of the alkanols^22^. Thus, instead of inhibiting the channel mutant, application of alkanols potentiated the current amplitude. An alanine substitution for the corresponding proline (P475) in *Shaker*-IR shifted the threshold for channel opening towards more depolarized potentials by affecting the late step(s) of channel gate opening while leaving earlier VSD transitions unaffected^29^. Consequently, the *Shaker*-IR-P475A mutant displays slow I_Kac_ kinetics that is only weakly voltage-dependent.

**Figure 7:**
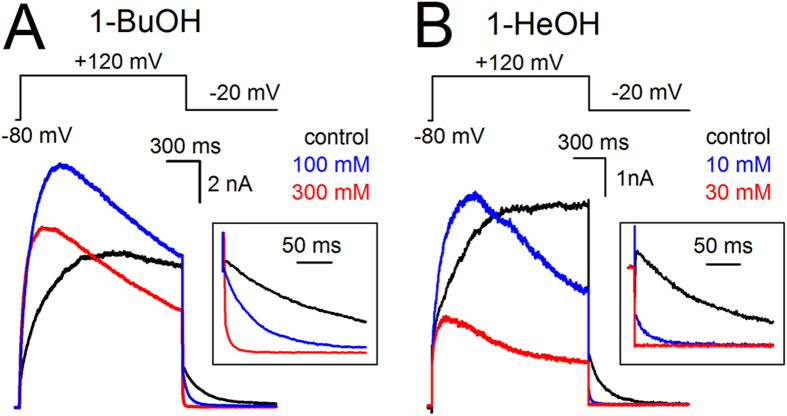
Alkanol-dependent activation of *Shaker*-IR-P475A. (**A**) Steady-state I_K_ recordings of *Shaker*-IR-P475A in control condition (black), 100* *mM (blue), and 300* *mM (red) 1-BuOH elicited using the pulse protocol shown on top. (**B**) Steady-state I_K_ recordings obtained in control condition (black), 10* *mM (blue) and 30* *mM 1-HeOH (red). In presence of alkanols the currents activated markedly faster and current inactivation was more pronounced. Insets show scale up views of the deactivating (I_Kdeac_) tail currents.

**Figure 8:**
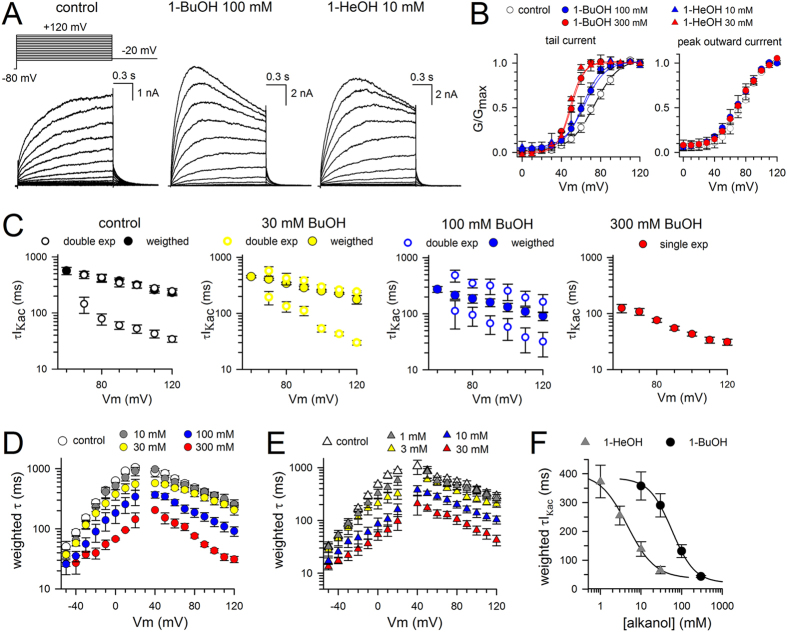
Biophysical properties of *Shaker*-IR-P475A upon alkanol application. (**A**) Representative I_Kac_ recordings of *Shaker*-IR-P475A in control conditions, 100* *mM 1-BuOH and 10* *mM 1-HeOH, elicited using the pulse protocol shown on top. (**B**) Conduction vs. voltage GV curves of *Shaker*-IR-P475A in control conditions (white circles), 100* *mM 1-BuOH (blue circles), 300* *mM 1-BuOH (red cricles), 10* *mM 1-HeOH (blue triangles), and 30* *mM 1-HeOH (red triangles). GV curves displayed in the left panel were obtained by normalizing tail current amplitudes. Solid lines represent the average fit with a Boltzmann equation (V_1/2_ and slope factor values are provided in Table 1). Right panel displays the GV curves determined from analyzing the peak outward currents. (**C**) Panels from left to right show the τI_Kac_ values of *Shaker*-IR-P475A upon increasing 1-BuOH concentrations with the left most panel showing the values in control conditions. The fast and slow τI_Kac_ components are represented with open symbols and the weighted τI_Kac_ with filled symbols. Note that the contribution of the fast τI_Kac_ component increased upon higher 1-BuOH concentrations; compare weighted τI_Kac_ values in 30* *mM (yellow symbols) and 100* *mM (blue symbols). In presence of 300* *mM 1-BuOH (red symbols) only the fast component could be resolved and I_Kac_ was approximated with a single exponential function. (**D**) Plot shows the weighted τI_Kac_ and τI_Kdeac_ values in control conditions (white) and in presence of 10* *mM (gray, *n* = 4), 30* *mM (yellow, *n* = 5), 100* *mM (blue, *n* = 8), and 300* *mM (red, *n* = 4) 1-BuOH. (**E**) Plot shows the effect of 1* *mM (gray, *n* = 7), 3 mM (yellow, *n* = 9), 10* *mM (blue, *n* = 5), and 30* *mM (red, *n* = 5) 1-HeOH on the weighted τI_Kac_ and τI_Kdeac_ kinetics. (**F**) Concentration-response curves obtained by plotting the weighted τI_Kac_ at +100* *mV as a function of 1-BuOH (black circles, *n* = 10) and 1-HeOH (gray triangles, *n* = 6) concentration. Solid lines represent the fit with a Hill equation.

Applying 1-BuOH or 1-HeOH to the *Shaker*-IR-P475A mutant resulted in a concentration-dependent increase in I_K_ and an acceleration of τ I_Kac_ (Fig. 7A,B), which is in agreement with previous data obtained in the *Shaw2* channel^22^. With higher concentrations of 1-BuOH or 1-HeOH the typical conduction versus voltage GV curves, which were determined from normalizing the deactivation tail current of activation protocols (Fig. 8A), appeared to become steeper and to shift slightly towards more hyperpolarized potentials (Fig. 8B, Table 1). However, concomitantly with the accelerated τI_Kac_ kinetics, also the inactivation process became more pronounced and the peak I_K_ amplitude started to decrease at higher alkanol concentrations (Fig. 7A,B). Therefore, the small hyperpolarizing shift and steepening of the GV curves could be an apparent effect due to the accelerated channel inactivation. To test this possibility, we determined the normalized conduction G from the peak outward currents using the Goldman-Hodgkin-Katz current equation. The GV curves obtained with this approach, which should be less sensitive to inactivation, were in presence of alkanols similar to those in control conditions (Fig. 8B). Thus, although both compounds resulted in I_K_ activation, neither 1-BuOH nor 1-HeOH affected the voltage dependence of channel opening substantially. To evaluate if the pronounced channel inactivation behavior reflects in fact open channel block, we examined I_Kdeac_ more closely. In contrast to what is expected with open channel block, the I_Kdeac_ recordings did not cross nor did they display a noticeable hook (Fig. 7A,B). In fact, the τI_Kdeac_ kinetics accelerated markedly which suggested that also the accelerated channel inactivation was due to gating modification. All these effects were fully reversible upon wash-out of both alkanols.

The I_Kac_ of *Shaker*-IR-P475A displayed two components and was best approximated with a double exponential function yielding a fast and a slow τI_Kac_ component^29^. However, the fast component contributed only marginally to the overall I_K_ amplitude and the weighted τI_Kac_ kinetics approximated the value of the slow component in control condition (Fig. 8C). 1-BuOH or 1-HeOH accelerated channel opening markedly but approximating the I_Kac_ currents with a double exponential function indicated that the time constants of both the fast and slow component were similar to those obtained in control condition. However, the contribution of the fast component in the total current amplitude increased as a function of alkanol concentration (Fig. 8C). Consequently, the weighted τI_Kac_ accelerated with increasing alkanol concentration (Fig. 8D,E). Similar to I_Kac_, the weighted τI_Kdeac_ kinetics, obtained from fitting I_Kdeac_ with a double exponential function, accelerated in an alkanol concentration-dependent manner (Fig. 8D,E). Plotting the weighted τI_Kac_ as a function of 1-BuOH or 1-HeOH concentration yielded concentration-response curves with IC_50_ values of 58.8 ± 3.0 mM (*n* = 5) and 4.6 ± 0.8 mM (*n* = 4), and Hill coefficients of 1.5 ± 0.4 and 1.3 ± 0.3, respectively (Fig. 8F). This indicated that the alanine substitution for P475 in S6_c_ did not affect the affinity for alkanols, suggesting that the conformation of the binding site remained intact.

### 1-BuOH and 1-HeOH did not affect the VSD movements of the P475A mutant

**Figure 9:**
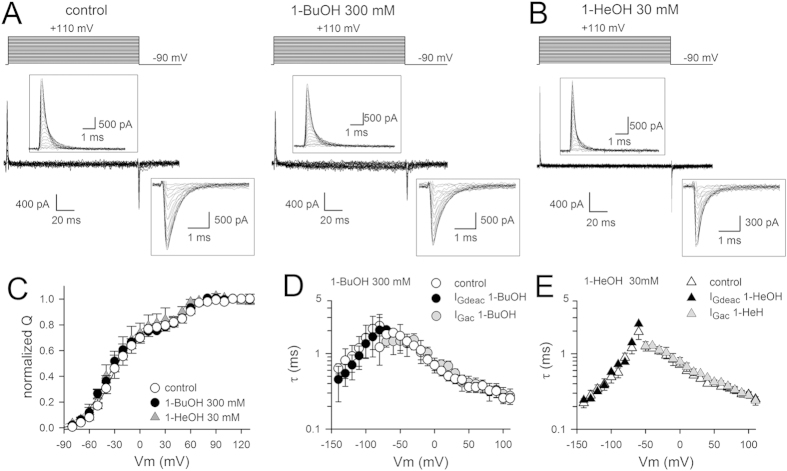
Alkanols did not affect the I_G_ behavior of *Shaker*-IR-W434F-P475A. (**A**) Representative I_G_ recordings of *Shaker*-IR-W434F-P475A recorded in control condition (left recordings) and after approximately 10 minutes wash-in of 300* *mM 1-BuOH (right recordings), elicited with the pulse protocols shown on top. Insets show scale up views of I_Gac_ and I_Gdeac_ respectively. (**B**) Representative I_G_ recordings of *Shaker*-IR-W434F-P475A in presence of 30* *mM 1-HeOH. (**C**) QV curves of *Shaker*-IR-W434F-P475A in control conditions (white circles) and presence of 300* *mM 1-BuOH (black circles, *n* = 5) or 30* *mM 1-HeOH (gray triangles, *n* = 4). (**D**) τI_Gac_ (gray circles) and τI_Gdeac_ (black circles) kinetics of *Shaker*-IR-W434F-P475A in presence of 300* *mM 1-BuOH, which were similar to the kinetics in control condition (white circles). (**E**) Similar to 1-BuOH, 30* *mM 1-HeOH did not affect the τI_Gac_ (gray triangles) or the τI_Gdeac_ (black triangles) kinetics compared to control condition (white triangles).

Since the *Shaker*-IR-P475A mutant did not affect the early VSD movements, the QV curve was split and displayed two gating charge components whereby the late one corresponded with the voltage dependence of channel gate opening^29^. Analyzing the gating currents of *Shaker*-IR-W434F-P475A in presence of 300 mM 1-BuOH or 30 mM 1-HeOH indicated that the voltage dependence of neither the early nor the late gating charge component was affected by 1-BuOH or 1-HeOH (Fig. 9 and Table 1). This was in agreement with the absence of an obvious shift in the threshold of channel opening (Fig. 8B). Also the I_Gac_ time constants, which in *Shaker*-IR-W434F-P475A report directly on the kinetics of the early VSD movements^29^, were unaffected by 1-BuOH or 1-HeOH. These I_G_ data confirmed that 1-BuOH and 1-HeOH did not affect the voltage-dependent transitions of the *Shaker*-IR-P475A mutant but facilitated a late largely voltage-independent transition in the activation pathway, a transition that is compromised by the P475A mutation.

## Discussion

1-BuOH and 1-HeOH inhibited the *Shaker*–IR channel in a concentration-dependent manner without displaying the classic hallmarks of an open channel blocker. Therefore, alkanols appear to act as gating modifiers that stabilize the channels in a non-conducting state^4^. To elucidate which state is stabilized by alkanols, we determined the impact of 1-BuOH and 1-HeOH on the I_G_ recordings of *Shaker*-IR-W434F. Both alkanols caused a concentration-dependent reduction in gating charge movement associated with accelerated VSD deactivation (Fig. 3). This data indicated that alkanols interfere with the transition from the non-conducting activated conformation to full channel gate opening, which occurs in a highly subunit-cooperative (concerted) manner. Consequently, the reduction in either I_K_ or gating charge movement as a function of alkanol concentration yielded concentration-response curves with similar IC_50_ values and Hill coefficients (Figs 1,3). Since alkanols have been proposed to operate via the S4-S5 linker^18^, there are 4 potential alkanol binding sites on the channel that appear to operate largely independently (Hill coefficients of approximately 1). As they interfere with a subunit-cooperative transition, binding of a single alkanol molecule (occupying only one out of four binding sites) can be sufficient to prevent channel gate opening and losing about 10% of the gating charge movement. Accordingly, a previous study, which used concatemeric constructs, showed that channels with less than four high affinity binding sites (e.g. only 2) were still inhibited by alkanols^4^. Thus, we propose that alkanols inhibit *Shaker*-IR currents by preventing the channels of passing the final concerted step in the activation sequence that opens the channel gate. Therefore, the ionic current data analysis represented in figure 2 reports on the channels that were free of alkanols which explains why both the normalized GV curves (Fig. 2C) and the kinetics (Fig. 2D) in presence of alkanols were similar to control conditions.

The impact of 1-BuOH and 1-HeOH on *Shaker*’s gating charge movement was reminiscent to that of the well-characterized drug 4-AP^14^, and both compounds stabilize the *Shaker* K_v_ channel in the non-conducting activated state. However, both compounds achieve this by acting via distinct binding sites (Fig. 6). Whereas the binding determinants for 4-AP, including those for guanidine compounds that possibly work in a similar manner^30^, reside within S6_c_^31^,^32^, alkanols are suggested to distort the coupling between the S4-S5 linker and S6_c_^33^. The observation that alkanols rescued partly the kinetics of the *Shaker*-IR-P475A mutant, favors the idea that alkanols alter the conformation of the S4–S5 linker and/or S6_c_ without disrupting their communication completely. By altering the conformation of the S4-S5 linker and/or S6_c_, the electromechanical coupling is compromised as its operation relies on a correct positioning of both segments with respect to each other^34^.

Mutations that affect the communication between the VSD and the channel gate might therefore alter the alkanol effect, as is the case in *Shaker*-IR-P475A. Apparently, 1-BuOH and 1-HeOH did not shift the voltage dependence of the late gating charge component in *Shaker*-IR-P475A (Fig. 9), which is expected if the mutation was only to affect the equilibrium constant of the transition from the non-conducting activated to the open state. Therefore, the structural consequences of the P475A mutation should be more severe and the *Shaker*-IR-P475A mutant displayed, accordingly, a biphasic current activation that in absence of alkanols is dominated by the slow component. We propose that alkanol binding to the *Shaker*-IR-P475A channel alters the conformation of the S4-S5 linker and/or its communication with S6_c_ (as it does in WT *Shaker*-IR), and in doing so it coincidentally restores the conformation of the S6_c_ channel gate that is compromised by the mutation. Alkanols then act as activators of the *Shaker*-IR-P475A mutant by yielding I_K_ current activation that is dominated by the fast component (Fig. 8C–E), thus accelerating a late largely voltage-independent transition of channel gate opening. Notably, the effect of both 1-BuOH and 1-HeOH on the *Shaker*-IR-P475A mutant was comparable to the behavior of poly-unsaturated-fatty acids (PUFAs): accelerating channel opening followed by more pronounced channel inactivation at higher concentrations (Fig. 7A,B). PUFAs have been shown to alter the kinetics of K_v_ channels leading to current activation or current inhibition, in part by accelerating the inactivation process^35^. At low concentrations several PUFAs act as channel activators but at higher concentrations they result in channel inhibition^36^,^37^,^38^. Whereas their activating property is ascribed to their ability to shift the voltage dependence of channel opening towards more hyperpolarized potentials and to facilitate the late subunit-concerted transition of channel opening^39^,^40^, their molecular mechanism to induce channel inhibition is still debated^41^. Whereas alkanols most likely target the S4-S5 linker of K_v_ channels^33^, PUFAs supposedly exert their effect through the VSD^39^,^40^, although a role for the S4-S5 linker has been suggested^42^.

Alkanols and 4-AP immobilize the same VSD movement(s) in the *Shaker*-IR channel (Fig. 5), but both compounds achieve this via distinct drug binding sites (Fig. 6) and a different mechanism of action. This conclusion is further supported by the observation that the mutant *Shaker*-IR-P475A is activated by alkanols (Figs 7,8) but is insensitive to 4-AP^29^. The presence of other (possibly overlapping) intracellular or lipid-accessible binding sites for gating modifying compounds is supported by: (1) the finding that the gating modifier toxin gambierol occupies a lipid exposed S5-S6 crevice outside the K^+^ pore^43^, a binding site which is most likely shared by psora compounds^44^ and (2) the observation that ruthenium complexes uncouple VSD movement from channel gate opening but in contrast to alkanols they immobilize about 50% of the gating charge^45^. Furthermore, the binding site for the volatile anesthetic halothane has been shown to overlap with that of alkanols^21^, and both isoflurane and servoflurane, which belong to the same class of halogenated general anesthetics, potentiate K_v_ channels instead of inhibiting them^46^,^47^,^48^.

The intoxicating and sedating effects of exposure to high alkanol concentrations are well described and ion channels (including K_v_ channels) do most likely form one of their molecular targets. We provide a mechanistic basis for understanding their effect on K_v_ channels and show that 1-BuOH and 1-HeOH interfere directly with the gating apparatus of the *Shaker*–IR K_v_ channel. They inhibit *Shaker*-IR by stabilizing the non-conducting activated state preventing the channels from passing the final subunit-concerted transition leading to channel gate opening. They achieve this through a unique gating modifying mechanism different from that of 4-AP. Our findings strengthen the idea that there exist different intracellular drug binding sites that via distinct mechanisms of action exert a similar gating modifying effect; this opens new possibilities for designing modulators of K_v_ channels.

## Methods

### Molecular Biology

The N-terminal deletion Δ6–46 *Shaker* clone (*Shaker*-IR), which removes fast inactivation^49^, was used in this study. The W434F mutation, which yields a non-conducting *Shaker*-IR-W434F channel^25^, and the P475A mutation were introduced as described previously^29^. All channel constructs were expressed using a pGW1 expression vector. The plasmid that codes for the green fluorescent protein, used to identify transfected cells, was purchased from Clontech (Palo Alto, CA, USA). Plasmid DNA for mammalian expression was obtained by amplification in XL2 Bluescript cells (Stratagene), and afterwards isolated using the endotoxin-free Maxiprep kit (Macherey-Nagel, Düren, Germany). The cDNA concentration was determined by UV absorption.

### Cell culture

HEK293 cells were cultured in Modified Eagle’s Medium (MEM) supplemented with 10% fetal bovine serum, 1% penicillin/streptomycin and 1% non-essential amino acids (Invitrogen, Carlsbad, CA, USA). Cells were transiently transfected with the appropriate channel DNA plasmids using polyethyleneimine that was purchased from Sigma-Aldrich (St Louis, MO, USA), details of procedure was described previously^29^.

### Electrophysiology

Whole-cell ionic I_K_ or gating I_G_ current measurements were done at room temperature (20 to 23 °C) using an Axopatch-200B amplifier and the recordings were digitized with a Digidata-1200 A acquisition system (Molecular Devices, Sunnyvale, CA, USA). Both I_K_ and I_G_ recordings were digitized at 10 kHz sampling rate after passing a 5 KHz Bessel low-pass filter. Command voltages and data storage were controlled with pClamp10 software. Patch pipettes were pulled from 1.2 mm quick-fill borosilicate glass capillaries (World Precision Instruments, Sarasota, FL, USA) with a P-2000 puller (Sutter Instrument Co., Novato, CA, USA) and afterwards heat-polished, to have patch pipettes with a resistance of approximately 1.5 MOhm determined with the filled pipette in the bath solution.

For I_K_ measurements the cells were constantly superfused with external bath solution that contained (in mM) NaCl 130, KCl 4, CaCl_2_ 1.8, MgCl_2_ 1, HEPES 10, Glucose 10, adjusted to pH 7.35 with NaOH. The patch pipettes were filled with internal solution containing (in mM) KCl 110, K_4_BAPTA 5, K_2_ATP 5, MgCl_2_ 1, HEPES 10, adjusted to pH 7.2 with KOH. For I_G_ measurements the monovalent cations were replaced with N-methyl-D-glucamine (NMG^+^). The bath solution contained (in mM) NMG^+^ 140, HEPES 10, Glucose 10, MgCl_2_ 1, CaCl_2_ 1.8, titrated to pH 7.35 with HCl. The pipette solution contained (in mM) NMG^+^ 140, HEPES 10, EGTA 10, MgCl_2_ 1, titrated to pH 7.2 with HCl. Junction potentials were zeroed with the filled pipette in the bath solution and experiments were excluded from analysis if the voltage error estimate exceeded 5 mV after series resistance compensation. For I_G_ measurements, leak currents and remaining capacitive currents were subtracted online using a −P/6 protocol (using a holding potential of −95 mV). I_K_ recordings were not leak corrected.

### Drug solutions

1-BuOH and 1-HeOH (Sigma-Aldrich, St. Louis, MO, US) were directly dissolved in the external recording solution for either I_K_ or I_G_ measurements. The different test concentrations were daily made as both compounds are volatile lowering the effective concentration upon storage. For the highest concentration of 1-BuOH tested (300 mM), the osmolarity of the extracellular solution for I_K_ or I_G_ recordings increased by approximately 300 mOsm resulting in a total osmolarity of ~640 mOsm. Because of the rapid partitioning of alkanols across the plasma membrane, we expected a minor impact of this increase in osmolarity. Indeed, the cells tolerated remarkably well the perfusion of the 300 mM 1-BuOH solution. This was not the case when the cells were perfused with a 600 mOsm extracellular solution that contained glucose, which does not easily partition across the plasma membrane, to increase osmolarity (data not shown). 4-AP was purchased from Sigma-Aldrich and after dissolving it in the external recording solutions the pH was adjusted to 7.35 using HCl. All compounds were applied to the cells using a pressurized fast perfusion system equipped with a quartz micromanifold (ALA scientific, Farmingdale, NY, USA), allowing rapid exchange of the external solutions.

### Data analysis

Details of pulse protocols used to elicit I_K_ or I_G_ recordings were adjusted to determine the biophysical properties of each construct adequately and are shown in the figures or described in legends. All the graphs were built using SigmaPlot 11.0 (Systat Software Inc., San Jose, CA, USA). If not mentioned otherwise, the conductance vs. voltage (GV) curves were determined from analyzing normalized tail current amplitudes and the charge vs. voltage (QV) curves by integrating the activating I_G_ currents. The QV and GV curves of *Shaker*-IR were fitted with a Boltzmann equation: *y* = 1*/*{1 + exp[−(*V* − *V*_1*/*2_)*/k*]}, where *V* represents the applied voltage, *V*_1*/*2_ the midpoint potential at which 50% of the total charge has moved or half of the channels have opened, and *k* the slope factor. For the P475A mutant the GV curve was also approximated with a single Boltzmann equation whereas its QV curve was approximated with the sum of two Boltzmann distributions. Activation I_K_ kinetics (τI_Kac_) were determined by approximating the rise in I_Kac_ with a single or double exponential function. Deactivation I_K_ kinetics (τI_Kdeac_) were obtained from single or double exponential fits to the I_Kdeac_ decay elicited at various repolarizing potentials following a 25 ms depolarizing pre-pulse to + 20 mV that activated the channels. When a double exponential function was used to determine the fast (τ_fast_) and slow (τ_slow_) component of the τI_Kac_ and τI_Kdeac_ kinetics, the weighted time constants (τ_W_) were calculated based on the amplitude of each component: τ_W_ = (A_fast_/(A_fast_ + A_slow_)) x τ_fast_ + (A_slow_/(A_fast_ + A_slow_)) x τ_slow_, with A_fast_ and A_slow_ the amplitude of the fast and slow component respectively. The I_G_ activation and deactivation kinetics (τI_Gac_ and τI_Gdeac_) were determined by fitting the decaying part of I_Gac_ and I_Gdeac_ with a single exponential function. All results are expressed as mean ± S.E.M. with *n* the number of cells analyzed.

Concentration–response curves (both from I_K_ and I_G_ analysis) were fitted in the program OriginPro 8 (OriginLab Corp., Northampton, MA, USA) with a Hill equation: I_effect_ = I_min_ + ({I_max_ − I_min_}/{1 + ([alkanol]/*IC*_*50*_)^*Hill coefficient*^}), where [alkanol] is the concentration of 1-BuOH or 1-HeOH and IC_50_ the concentration that induces 50% effect. To test whether 1-BuOH shares a similar and/or overlapping binding site with 4-AP, we performed competition experiments based on a previously described approach^28^. The method is based on comparing the experimental determined inhibition to the expected level of channel inhibition using an allotopic (non-competing) or a syntopic model (competing). Formulas used for calculating the expected inhibition in presence of both compounds (*I*_*NX,Y*_) according to the allotopic and syntopic model were *I*_*NX,Y*_* = (I*_*NX*_ *+ I*_*NY*_ − *I*_*NX*_*I*_*NY*_) and I_*N*X,Y_ = ((*I*_*NX*_ *+ I*_*NY*_ − *2I*_*NX*_*I*_*NY*_)/(1− *I*_*NX*_*I*_*NY*_)), respectively. *I*_*NX*_and *I*_*NY*_ were the experimentally determined level of channel inhibition induced by each compound independently. I.e. *I*_*NX*_ was the inhibition induced by 1-BuOH and *I*_*NY*_ the level of inhibition induced by 4-AP. A two way analysis of variance (ANOVA) was used to determine the differences of a dual inhibition. A *post hoc* Dunnett’s was used to compare both models.

## Additional Information

**How to cite this article**: Martínez-Morales, E. *et al.* Alkanols inhibit voltage-gated K^+^ channels via a distinct gating modifying mechanism that prevents gate opening. *Sci. Rep.* **5**, 17402; doi: 10.1038/srep17402 (2015).

## Figures and Tables

**Table 1:** Midpoint (V_1/2_) and slope factor (*k*) values of the GV and QV curves of *Shaker*-IR, *Shaker*-IR-P475, and their non-conducting W434F variants.

	GV curve *(voltage dependence of gate opening)*				
*Shaker*-IR	**V**_**1/2**_ **(mV)**	***k*****(mV)**			***n***
control conditions	−27.0 ± 1.3	5.6 ± 0.7			11
1-BuOH (50* *mM)	−28.5 ± 2.3	5.1 ± 0.7			5
1-HeOH (3 mM)	−25.4 ± 1.2	7.5 ± 0.9			6
*Shaker*-IR-P475A	**V**_**1/2**_ **(mV)**	***k*****(mV)**			***n***
control conditions	74 ± 1	12.3 ± 0.7			8
1-BuOH (300* *mM)	50 ± 1	6.8 ± 0.7			4
100* *mM	62 ± 1	9.6 ± 0.4			8
1-HeOH (30* *mM)	49 ± 1	6.6 ± 0.6			5
10* *mM	64 ± 1	10.7 ± 0.6			5
					
	**QV curve** *(voltage dependence of VSD movement)*				
*Shaker*-IR-W434F	**V**_**1/2**_ **(mV)**	***k*****(mV)**			***n***
control conditions	−33.2 ± 5.4	−11.6 ± 2.0			10
1-BuOH (300* *mM)	−32.4 ± 3.4	−12.5 ± 1.7			5
1-HeOH (30 mM)	−31.4 ± 0.9	−10.1 ± 0.8			4
					
	***1st comp***		***2nd comp***		
*Shaker*-IR-W434F-P475A	**V**_**1/2**_ **(mV)**	***k*****(mV)**	**V**_**1/2**_ **(mV)**	***k*****(mV)**	***n***
control conditions	−33.1 ± 0.8	14.5 ± 0.8	59.6 ± 2.7	10.1 ± 2.4	8
1-BuOH (300 mM)	−39.2 ± 1.4	16.2 ± 1.2	52.2 ± 1.2	9.1 ± 1.1	5
1-HeOH (30 mM)	−35.3 ± 1.5	16.9 ± 1.5	50.2 ± 1.1	3.6 ± 1.4	4
					
